# Does HIV/AIDS Funding Undermine Health Systems?

**DOI:** 10.4269/ajtmh.2012.12-0291a

**Published:** 2012-09-05

**Authors:** Victoria A. Fan, Rachel Silverman, Amanda Glassman

**Affiliations:** Center for Global Development; Washington, District of Columbia; E-mails: vfan@cgdev.org, rsilverman@cgdev.org, and aglassman@cgdev.org

Dear Sir:

We read with interest the recent article by Shepard and others,^1^ which attempted to evaluate the impact of human immunodeficiency virus/acquired immunodeficiency syndrome (HIV/AIDS) funding on Rwanda's health system. The headline of the associated press release is assertive: “Six-Year Study in Rwanda Finds Influx of HIV/AIDS Funding Does Not Undermine Health Care Services for Other Diseases. Study Addresses Long-Standing Debate about Funding Imbalances for Global Diseases.”^2^ However, the study's results are far from a definitive answer to the policy question of whether AIDS funding has undermined or enhanced efforts on non-AIDS service provision.

The main threat to the validity of this study relates to the assignment of treatment of HIV/AIDS funding to health centers. In 2008, Shepard and others^1^ collected retrospective data on the performance of 25 health centers that received HIV/AIDS funding between 2002 and 2007. These health centers are said to be “randomly selected as the intervention group” by Shepard and others^1^ and then “perfectly matched” to 25 other health centers serving as control units.^1^ Unfortunately, the intervention group was “randomly” selected only in the sense that Shepard and others^1^ chose to study them, not that the health centers in the intervention group were randomly assigned to treatment. Without random assignment and appropriate strategies for causal inference, there is likely to be treatment imbalance error, which arises from observable and unobservable differences between intervention and control centers. The non-random assignment of the intervention group compromises the internal validity of the study and its assertions on the causal inference regarding the effect of the intervention (AIDS funding to health centers) on the outcomes (performance on non-HIV/AIDS service delivery indicators).

A serious problem that the work by Shepard and others^1^ faces is that it is unclear, if not unknown, why treatment was assigned to those 25 centers in the first place. Even if Shepard and others^1^ had enough money to get data for all the HIV/AIDS health centers and did not have to take a random subset of 25 HIV/AIDS centers, the work by Shepard and others^1^ needs to address why these health centers were chosen to receive HIV/AIDS funding to start. For example, it would not be surprising if the government assigned centers to receive HIV/AIDS funding because those centers were more likely to have better outcomes, or were believed to be more successful or capable before treatment assignment. Such beliefs could stem from the unmeasured characteristics of ability and leadership of those centers, their political connections, or even potentially measurable characteristics such as better (or better paid) doctors in those facilities. The centers may also have been chosen because they serve a larger population and could scale up more quickly, or for an array of other factors that might also affect the outcomes of interest. To strengthen the study, the work by Shepard and others^1^ needs to explain in detail how the intervention units were actually assigned by policymakers or the government and control for these factors in order to reduce the bias of their estimates.

Moreover, the work by Shepard and others^1^ needs to provide data on other pre-treatment variables to offer assurance that the intervention group was not different from the control group before the intervention began, even if such variables are not ultimately included in the multiple regressions. To give some credit, the work by Shepard and others^1^ attempted to make the intervention group similar to the control group by matching the 25 intervention health centers to 25 control health centers–this matching is important. Matching after assignment of treatment can help to achieve balance on observables.^3^ However, the work by Shepard and others^1^ matches only on three characteristics: (1) health center ownership, (2) performance-based financing, and (3) district income in 2002. This assessment might be reasonable if these were the only three factors on which the treatment was assigned, and if there were no other differences between intervention and control groups that affected the outcomes (e.g., the factors mentioned above). However, without an explanation of the treatment assignment, it is not clear that these were the criteria for assignment as an AIDS center. In addition to these three matching variables, the work by Shepard and others^1^ also included three additional variables to reduce some confounding bias: a proxy for community-based health insurance coverage, accessibility of the health center by bus, and the background of the health center's director. Although the work by Shepard and others^1^ had a limited sample size and statistical power, it is likely that the intervention and control groups differed beyond these six characteristics (for example, by the number of providers and the demographic and epidemiological characteristics of the catchment population among others) and hence not “perfectly matched.” Even simple *t*-tests on the difference in means between intervention and control groups for each plausible confounding variable would be a minimal starting place and could be slightly reassuring. However, using such *t*-tests is not a sufficient or exhaustive means to check balance in matched samples.^3^ Even better, Shepard and others^1^ could calculate a new multivariate imbalance measure that captures multidimensional imbalance between intervention and control groups beyond mean imbalance.^4^

Without verifying at least these issues through additional checks, the findings of this study for Rwanda are not convincing. These checks, however, should not be too difficult if Shepard and others^1^ have additional unpublished data (e.g., the data that they used to match the intervention group to the control group), or if they collected more facility characteristics beyond these three or six variables before matching or used publicly available data from the Demographic and Health Surveys^5^ or the 2002 Rwandan Census.^6^ The study would benefit greatly from additional checks and qualification of their findings because of potential bias arising from treatment imbalance.
